# Morphological and Biological Evaluations of Human Periodontal Ligament Fibroblasts in Contact with Different Bovine Bone Grafts Treated with Low-Temperature Deproteinisation Protocol

**DOI:** 10.3390/ijms23095273

**Published:** 2022-05-09

**Authors:** Serena Bianchi, Sara Bernardi, Antonella Mattei, Loredana Cristiano, Leonardo Mancini, Diana Torge, Giuseppe Varvara, Guido Macchiarelli, Enrico Marchetti

**Affiliations:** 1 Department of Life, Health and Environmental Sciences, University of L’Aquila, 67100 L’Aquila, Italy; serena.bianchi@univaq.it (S.B.); antonella.mattei@univaq.it (A.M.); loredana.cristiano@univaq.it (L.C.); leonardo.mancini@graduate.univaq.it (L.M.); diana.torge@graduate.univaq.it (D.T.); guido.macchiarelli@univaq.it (G.M.); enrico.marchetti@univaq.it (E.M.); 2 Center of Microscopy, University of L’Aquila, 67100 L’Aquila, Italy; 3 Department of Innovative Technologies in Medicine and Dentistry, University of Chieti-Pescara ‘Gabriele d’Annunzio’, Via Dei Vestini 11, 66100 Chieti, Italy; gvarvara@unich.it

**Keywords:** biomaterial, cell culture, deproteinised bovine bone, fibroblasts, periodontal regeneration

## Abstract

Several types of deproteinised bovine bone mineral (DBBM) are available on the market, and each one is obtained with a thermic and chemical process that can differ, achieving different results. Currently, several protocols using low temperature are suggested to reduce the possible particle crystallisation during the production process. This study aimed to evaluate the biomorphological reaction of periodontal fibroblast cultures in contact with different DBBM particles treated with a low-temperature protocol (Thermagen^®^) and without exposure to sodium hydroxide (NaOH). Morphological evaluation was performed using light, confocal laser, and scanning electron microscopy, and the biological reaction in terms of proliferation was performed using an XTT proliferation assay at 24 h (T1), 72 h (T2), and 7 days (T3). The morphological analysis highlighted how the presence of the materials stimulated a change in the morphology of the cells into a polygonal shape, surface reactions with the thickening of the membrane, and expression of actin. In particular, the morphological changes were appreciable from T1, with a progressive increase in the considered morphological characteristics at T2 and T3 follow-ups. The proliferation assay showed a statistical significance between the different experimental materials and the negative control in T2 and T3 follow-ups. The post hoc analysis did not reveal any differences between the materials. In conclusion, the grafts obtained with the low-temperature extractions protocol and not exposed to NaOH solution showed positive morphological reactions with no differences in the sizes of particles.

## 1. Introduction

Regenerative procedures have received much interest in recent decades due to the demand for restoration and regeneration techniques of missing tissues and organs [1]. In the dental field, several techniques have been proposed to increase bone or soft tissue around teeth, implants, or for future prosthetic rehabilitation [2,3,4]. Early on, autologous biomaterials were the elective choice for treating periodontal defects and bone regeneration [5,6]. Autologous grafts are still the gold standard due to their capacity for osteoinduction and osteogenesis [7]. However, limited availability, a second surgical site, and discomfort for the patients limit the use of these biomaterials [6,8]. Thus, several biomaterials have been proposed and are constantly in development. As reported in the literature, there are several bone substitutes: autologous, allogeneic, xenogeneic, and synthetic [9,10].

As mentioned before, autologous is the gold standard, and it is harvested from intraoral (chin symphysis, mandibular ramus) or extraoral sites (iliac crest, calvaria) [11,12,13]. The advantages are biocompatibility, osteoinduction, and regenerative potential due to the presence of active osteoblasts and osteoclasts (osteogenesis) [6]. Allografts are bone grafts extracted from a subject of the same species. The advantages are osteoconduction and osteoinduction and availability, while the limitations are biocompatibility, possible transmission of diseases, such as the human immunodeficiency virus (HIV), and spongiform encephalopathy [14].

**Figure 1:**
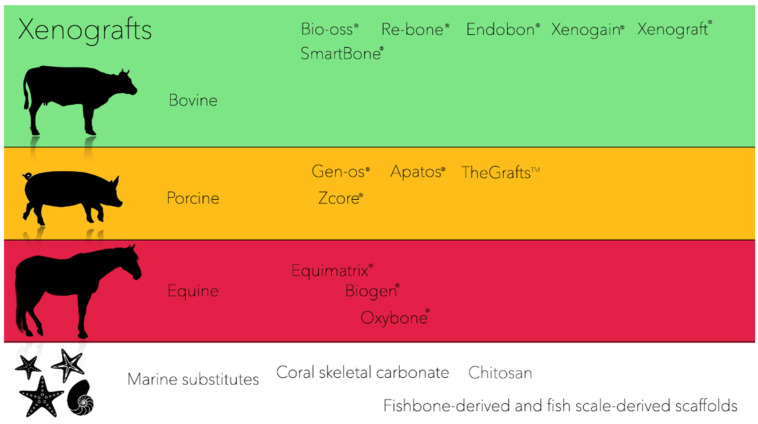
Examples of xenografts substitutes on the market.

Xenografts are most commonly used and diffused for oral regeneration, mainly for periodontal, peri-implant, and bone regeneration [15], owing to their osteoconduction properties. However, xenografts are commonly harvested from other species and usually derive from bovine, porcine, or equine origin (Figure 1) [9]. In addition, another source of xenografts is represented by marine substitutes (e.g., coral skeletons, fish bones, etc.), which showed potential osteoconduction properties beneficial to periodontal and bone regeneration [10].

In particular, periodontal regenerative procedures exploit multiple approaches and use combined biomaterials (grafts and autologous platelet concentrates) to provide the best outcomes [16,17,18,19]. Among the xenograft derived from mammals, the most commonly used is deproteinised bovine bone mineral (DBBM). The chemical process common for treating these substitutes is based on thermic oscillation. The primary aim is to remove the organic part of the tissue and bacteria and viruses to reduce the possible transmission of disease [14]. More precisely, the grafts are subjected to a temperature of 300–900 °C and a chemical process, which includes exposure to sodium hydroxide (NaOH), to remove the organic material [20]. The architecture, composed mainly of hydroxyapatite, similar to human bones, is preserved [21,22].

The thermal process was the primary choice, and several studies associated a high temperature with better macro- and microporosity of the grafts [20]. According to the literature, the optimal temperature is 300 °C for a well-represented microporosity structure [21,22]. This temperature is able to influence the inorganic structure of the biomaterial with cracks formation and the occurring of a ceramisation process, which can affect the presence of hydroxyapatite in the grafts [23]. Thus, in recent decades, several companies have evaluated the possible use of a low-temperature treatment for preserving the structure and chemical composition [24]. Beyond the thermal process, the available grafts are submitted to chemical treatment with NaOH, making hydroxyapatite bone porous chip material [25]. With research on new, sustainable, and better performing biomaterial, new manufacturing protocols have been developed and proposed. In particular, this new protocol includes a low-temperature treatment to prevent the formation of cracks and to preserve bone architecture. This study aimed to investigate how human periodontal ligament fibroblasts (HPLF) behave when in contact with DBBM, considering different compositions, granulometry, and low-temperature protocol of deproteinisation and NaOH exposure, assessing the proliferation and any cellular morphological changes.

## 2. Results

The combined analyses of XTT assay proliferation and morphological observations allowed a full and integrated overview of the performance of the examined DBBM material in contact with HPLF.

### 2.1. Proliferation Assay

**Figure 2:**
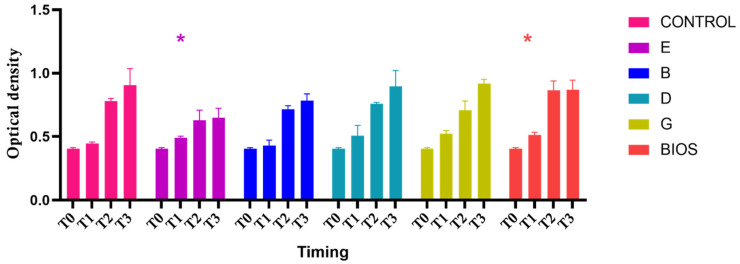
Separated graph bar showing the grouped data of mean and SD of the optical density values (*Y*-axis) of the cell not exposed to materials (control), and exposed to the different grafts (B, D, G, E, and BIOS) at the different timings (T0, T1, T2, and T3). * indicates the two-way ANOVA considering the variation in the OD to assess any difference absorbance differences in the considered materials between the group exposed to the experimental materials and the negative control at the different times of seeding (T0) and the T1, T2, and T3 follow-up resulted statistically significant.

In Figure 2 the graphs of proliferation assays showed a proliferation growth curve in the test materials (E, B, D, G), positive control (named as BIOS), and negative control (no material). The two-way ANOVA, considering the variation in the OD from the T0 and T1, T2, and T3 follow-ups, was statistically significant (*p* < 0.05). The post hoc Dunnett’s analysis showed a significant variation in the growth curve in the negative control and all experimental materials from the T0 and a significant growth difference between the negative control and the groups exposed to experimental group E and the positive control Bio-Oss (BIOS) at the T1 follow-up (Table 1). The post hoc Bonferroni analysis did not show any significant variation in the growth of the material at any follow-up.

### 2.2. Morphological Analysis—LM

**Figure 3:**
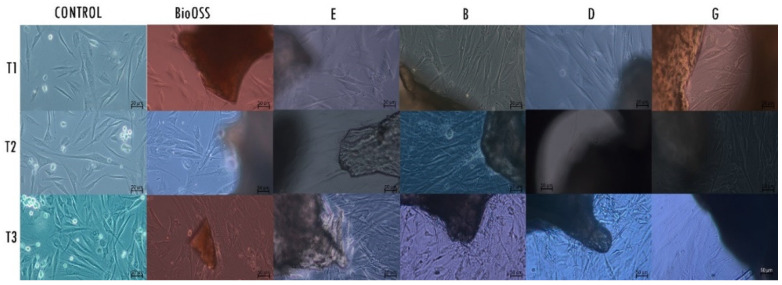
Contrast-phase light microscopy images of HPLF cells with the examined materials and the negative control at the different examined times; magnification 20×.

LM analysis showed a high-density layer of healthy fibroblasts in all groups of cells exposed to the materials, with differences between them (Figure 3). At the T1 timing of the culture, the control group of fibroblasts presented small dimensions and a fusiform shape of the cellular body. All experimental groups showed cells with a shape larger than that in the control group, presenting cytoplasmatic extensions in the proximity of the biomaterial. The positive control group also showed cells of larger dimensions. At T2, the shape of the cells of the negative control group continued to appear fusiform with opalescence signs. The group E cells appeared slightly fusiform, with cytoplasmatic prolongations towards the material. The group B cells appeared to be multilayer, with a high presence of cytoplasmatic processes in the proximity of the material. Groups D and G showed the presence of cells with enlarged cellular bodies. The cells exposed to the Bio-Oss graft presented a large shape with cytoplasmatic extensions. At T3, the negative control cells showed cells with fusiform morphology; the cells of the experimental groups E, B, D, and G, and cells of the positive control group Bio-Oss (BIOS) had a polygonal shape, were densely packed, and had cytoplasmatic extensions towards the biomaterials.

### 2.3. Morphological Analysis—SEM

**Figure 4:**
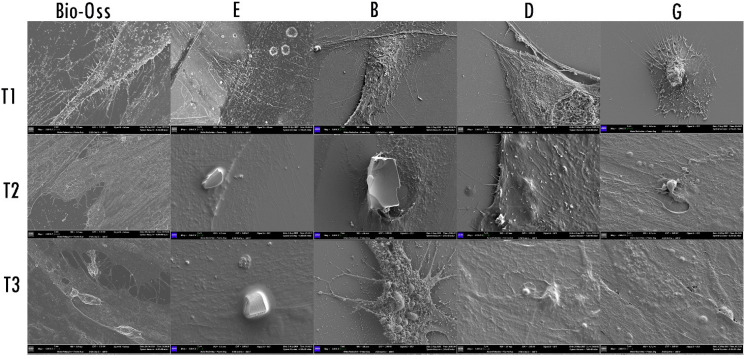
SEM images. Different reactions of the fibroblasts when exposed to the grafts Bio-Oss, E, B, D, and G, at 24 h, 72 h, and 7 days; magnification 500×.

The SEM observation (Figure 4) showed that, at T1, the body of fibroblasts exposed to biomaterials E, B, D, and G, and Bio-Oss appeared enlarged and with cytoplasmatic extensions and digitation on the membrane. At T2, the fibroblasts exposed to the E biomaterial appeared flat, while the cells exposed to experiment biomaterial B produced a cytoplasmatic extension towards the biomaterial; those exposed to biomaterial D continued their thickening, while those exposed to G appeared flat. Fibroblasts exposed to Bio-Oss appeared thicker, with cytoplasmatic extensions and digitation on the membrane. At T3, the fibroblast surfaces exposed to E biomaterial continued to appear flat and covered the bone particles. The surface morphology of the cells exposed to material B appeared highly dynamic and with the presence of thick cytoplasmatic prolongation. In contrast, the morphology of the fibroblast surface in contact with D and G biomaterial appeared flat but covered and incorporated the biomaterial particles. The cells exposed to Bio-Oss continued to appear thick, incorporating bone particles with cytoplasmatic prolongation from the membrane.

### 2.4. Morphological Analysis—CLSM

**Figure 5:**
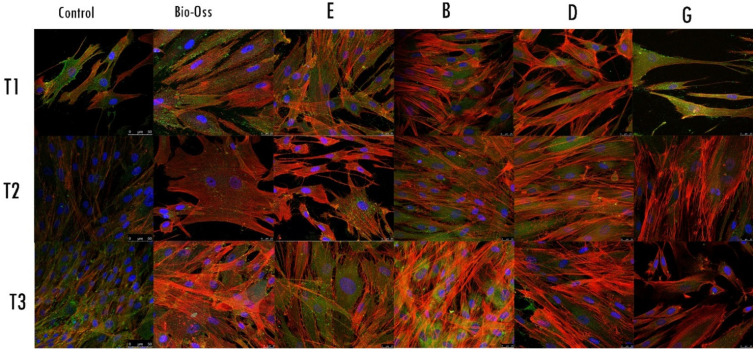
Confocal laser scanning microscopy (CLSM) images showing actin filaments using phalloidin in control and treated fibroblasts marked with CD90 (green), at different times of follow-up; magnification 63×.

The CLSM observations allowed us to assess the status of the nuclei (blue) and the expression and distribution of the actin (red). The nuclei showed an oval or rounded (blue) shape and were well represented in all samples. The actin (red) staining was different between groups and among the follow-up times. The negative control group showed weakness in the red signal; at the T3 follow-up, the signal slightly increased. At the T1 follow-up, experimental groups E, B, and D showed a strong red signal, which progressively increased at T2 and T3 follow-ups. The actin distribution was observed in the cellular contour and in the cellular projections and protrusions. Regarding the fibroblasts exposed to G, the material showed a weak actin signal at the T1 follow-up, mainly present at the contour of the body cells. The G samples started to express a stronger actin signal at T2, distributed in the cytoplasmatic projections; the signal and the distribution increased at the T3 follow-up. Regarding the sample Bio-Oss, the actin signal was expressed at the T1 follow-up and increased in T2 and T3 follow-ups (Figure 5).

## 3. Discussion

### 3.1. Xenograft Physiochemical Properties Given by the Manufacturing Process Play a Crucial Role in Cellular Stimulation

The quality of the regenerative properties of grafting materials relies on their physiochemical compositions, which influence the cellular conductivity and the induction and stability of the scaffold, and the degradation processes [26]. Beyond research exploring other alternatives, xenografts are still solid and frequent in oral surgery and periodontal regeneration procedures. One of the properties that are still under debate among researchers is the size of the granules. Several studies have investigated the effect of the size of the granules in bone tissue regeneration procedures [27,28]. Klüppel et al. [28] assessed how granules of small size (0.4 mm) are resorbed faster than large granules and lead to the formation of osteoid tissue. Prieto et al. [27] confirmed this using a different type of graft, assessing how the particles’ size and the properties’ surface influence the subsequent remodelling process. Similarly, Leiblein et al. [29] determined how the particle sizes of grafts affect the regenerative process in terms of bone formation and inflammation.

The quality of the collected bone particles also influences the regenerative process: cancellous bone grafts present a wide surface area with a trabecular structure, which can promote new vascular supply and graft integration [30]. On the other hand, this trabecular structure does not well support the load due to weak structural stability [30].

Cortico-cancellous bone instead gives more stability and resistance to compression loads beyond inducing cellular proliferation in the trabecular structures, as reported by Mazzoni et al. [31].

Due to their nature, treatments used to remove prions and animal protein from bovine-derived grafts were required. These treatments (high temperature and chemical processes) appear to influence the structure and surface of the biomaterials and their mineral content and stability over time. Indeed, xenografts submitted to higher temperatures result in products being slowly absorbable [32]. In 2017, Fernàndez et al. [33] compared the resorption process of two biomaterials in vivo, one subjected to low temperature and one to high temperature. The xenograft treated with low temperature presented a lower ratio of mineral phase (Ca/P), collagen, higher porosity, and a higher capacity to be degraded than the graft treated with high temperature [34]. However, in 2020, Block evaluated the stability of high-temperature biomaterial by using an animal origin over a long period of time and concluded that using this type of material in ridge augmentation did not guarantee long-term stability [34]. Interestingly, Gehrke et al. [35] found a low cellular proliferation of a pre-osteoblastic line exposed to both types of xenografts (one treated with high temperature and chemical solvents and the other not), with no differences at the T2 follow-up. In addition, Gehrke et al. [35] highlighted how the temperature treatment affected the porosity and, therefore, the cellular capacity of the material’s penetration, which affects the cellular viability tests.

Our study assessed the cellular reaction in terms of proliferation and morphology of HPLFs towards biomaterial of different sizes, not submitted to high temperature, composed of different types of bone with promising results. Indeed, the proliferation data showed positive growth in all follow-ups and experimental materials, with no difference between them, considering both the size and the composition. Beyond confirming the proliferative data, morphological analyses highlighted the qualitative differences in the observed in vitro culture. Furthermore, LM observations indicated modifications in the cellular shapes of all exposed samples. In contrast, SEM observations showed a progressive thickening of the membrane and the development of cellular projections and extension towards the biomaterial in those cells exposed to the cortical and cancellous granules with a size between 0.25 and 1 mm (sample B) and not exposed to chemical deproteinisation. The fibroblasts exposed to the other three samples, E (cancellous granules with a size between 0.25 and 1 mm), D (cortico-cancellous granules with a size between 0.25 and 1 mm), and G (cancellous granules with a size between 1 and 2 mm), showed a progressive flattening of the membrane and the development of external projections. In addition, SEM observations showed different cell culture behaviour when exposed to the positive control material (cancellous granules with a size between 0.25 and 1 mm exposed to high temperature and chemical deproteinisation), with a progressive thickening of the membrane in the considered follow-ups. CLSM images support SEM findings and stress how the actin signal appears stronger in B and D samples than in E and G samples in T1 and T2 follow-ups. Moreover, CLSM observations confirmed the stimulation of the membrane from the positive control material, given the progressive increase in the actin signal from T1 to T3.

The progressive increase in the actin signal and morphological changes in the cellular shape indicated how the xenografts stimulated a reorganisation of the cytosolic actin and cytoskeleton modifications. Therefore, the signal of actin protein, due to its role in shape modifications and migration, as well as cell organelle movements, can be considered a definitive marker of fibroblast activations [36].

### 3.2. Effects of Graft Chemical Deproteinisation on Cellular Morphology

Chemical treatment is another critical factor influencing the final properties of the product. The World Health Organisation (WHO) guidelines for prions’ inactivation recommended the use of NaOCl and NaOH [37]. Chemical deproteinisation is a fundamental step to assure the removal of prions and animal proteins, which might result in an immunological reaction by the host organism. However, the effects of different solvents on the properties of the graft must be considered. For example, in 2015, Lei et al. [38] reported how xenografts deproteinised with pepsin have more osteogenic properties than those submitted to H_2_O_2_ deproteinisation. Bi et al. [25] studied the cancellous bone treated with NaOH and determined how these xenografts presented surface fissures, high organic content of Ca and P, lower resistance to mechanical stress, low trabecular thickness, and low cytocompatibility.

The present study’s data agreed with those in the literature, as shown by the XTT proliferation assays and morphological observations. The Bonferroni post hoc analysis did not show any significant differences between the experimental materials submitted to the chemical process of deproteinisation. The LM morphological observations allowed us to visualise an increase in cellular body sizes and a modification into a polygonal morphology, confirmed by SEM observations showing the development of cellular projections towards experimental material B and supported by the expression of a strong actin signal in CLSM investigations. Apparently, the low temperatures and the lack of exposure to the chemical deproteinisation stimulated the cytosolic actin, development of lamellipodia, and a thickening of the cellular membrane, and, therefore, a possible cellular migration towards the biomaterials.

We can speculate that possible clinical implications of using xenografts processed with low temperatures and not chemically exposed in cases of periodontal defects regeneration are a faster healing process due to important conduction and stimulation at the cellular level.

### 3.3. Strengths and Limitations

These observations revealed cellular activation and interaction of the cells with the microenvironment and adhesion to the biomaterials. The interesting interaction between the surface chemical and physical properties of three different xenografts and HPLFs can be useful for future clinical studies using biomaterials not subjected to chemical exposure for deproteinisation.

Limitations of the study are represented by the comparisons of different and heterogeneous groups of biomaterials (different sizes and different compositions).

## 4. Materials and Methods

### 4.1. Biomaterials

Deproteinised bovine bone grafts were harvested from young cattle (24 months), and after the first treatment of graft preparation for particle size and shape, a phase of purification through a thermic process was carried out. Grafts were subjected to a thermic shock, in the first phase at a high temperature (121 °C), and at a low temperature (80 °C; called Thermagen^®^) in the second phase.

**Figure 6:**
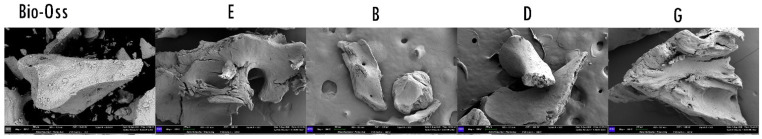
Scanning electron microscope (SEM) of bone grafts groups B, D, G, E, and Bio-Oss (BIOS). According to the manufacturing instructions, the bone grafts groups B and D are mainly cortical, and the bone grafts group G appears to be more cancellous; magnification 200×.

The grafts (Figure 6) were divided into the following four experimental groups:

- Group Bone E: cancellous granules with a size between 0.25 and 1 mm, exposed to NaOH for 1 h for deproteinisation;
- Group Bone B: cortical and cancellous granules with a size between 0.25 and 1 mm, not exposed to NaOH for deproteinisation;
- Group Bone D: cortical and cancellous granules with a size between 0.25 and 1 mm, exposed to NaOH for 1 h for deproteinisation;
- Group Bone G: cancellous granules with a size between 1 and 2 mm, exposed to NaOH for 1 h for deproteinisation;

As a positive control, a graft of cancellous granules was designed with a size between 0.25 and 1 mm and exposed to NaOH for 1 h for deproteinisation at a high temperature (Bio-Oss, named BIOS in the experiment; Figure 6).

### 4.2. Cell Culture

HPLFs were used and cultured as the manufacturer’s instructions suggested (ScienceCell Research Laboratories, Carlsbad, CA, USA). Initially, the first vial containing 5 × 105 cells in 1 mL of volume was cultured in four plastic culture dishes in a fibroblast medium (Innoprot. Derio, Bizkaia, Spain). The dishes were incubated under standard cell culture conditions of 37 °C in 5% CO_2_. The fibroblast medium was composed of a 500 mL basal medium, 2% foetal bovine serum (FBS), 1% fibroblast growth supplement, and 1% penicillin–streptomycin solution. Once subconfluence was reached, cells were detached using 0.05% trypsin (Innoprot. Derio, Bizkaia, Spain) and then subcultured at a density of 110 cells/mm^2^. Thus, the subculture passage used for the experimental phase was 10. HPLF is a cellular line characterised by the production of osteoblast-related extracellular matrix proteins, alkaline phosphatase activity, and active participation in inflammatory and immune-related events during periodontal diseases [39,40]. Due to the mentioned properties, and to evaluate the behaviour of periodontal cells in cases of use of xenografts in periodontal defects regeneration, the HPLFs model was chosen.

### 4.3. Cell Proliferation Assay and Statistical Analysis

The cell proliferation assay was performed according to the ISO EN 10993-5 standard. Briefly, 1 g of each sterile sample was placed into 24-well plates. Then, 1 mL of fibroblast medium was added (extraction medium) to each well and incubated for 24 h at 37 °C. Under standard cell culture conditions, 103 cells per well were seeded in a 100 µL extracted DMEM medium. The negative control group was seeded in a fibroblast medium. The XTT assay (Cayman Chemical, Ann Arbor, MI, USA) allowed observation of the cellular starting condition (T0) and proliferation activity at 24 h (T1), 72 h (T2), and 7 days (T3) follow-ups at an absorbance wavelength of 450 nm. XTT tests were performed with three technical replicates.

After assessing the normal distribution of the data, a two-way analysis of variance (ANOVA) test and Dunnett’s and Bonferroni’s post hoc analyses for multiple comparisons were performed to assess any significant variation between T0 and T1, T2, and T3 follow-ups.

Statistical analysis and graphs were performed using GraphPad Prism 9.1.1 (GraphPad Software, San Diego, CA, USA).

### 4.4. Morphological Analysis

A morphological evaluation of cells was performed using light microscopy (LM) to obtain a first-sight overview of the shape of the cultured cell. Scanning electron microscopy (SEM) was used to observe the cell membrane reaction, and confocal laser scanning microscopy (CLSM) was used to assess morphological changes in the cytoskeleton.

#### 4.4.1. Morphological Analysis—LM

Cells were seeded in 60 mm diameter plastic culture dishes with the test materials, control material, and without the material and incubated under cell culture conditions. At T1, T2, and T3 follow-ups, the dishes were observed using a phase-contrast light microscope (ZEISS Primovert, Jena, Germany), and a ZEISS Axiocam 208 colour camera was used to capture the images at 10× and 20×.

#### 4.4.2. Morphological Analysis—SEM

At T1, T2, and T3 follow-ups, the cells, seeded on a covered glass in 60 mm diameter plastic culture dishes containing the test materials or the control, were fixed using a 2% solution of glutaraldehyde, dehydrated in an ascending concentration ethanol solutions scale of 70, 80, and 90%, and three times at 100% for 10 min each. Afterwards, the samples were immersed for 3 min in 100% HDMS (Sigma-Aldrich S.r.l., Milan, Italy). Afterwards, the samples were air-dried by evaporation of hexamethyl-disilane (HDMS). After transferring the sample into a desiccator to prevent water contamination, the covered glasses were mounted on metal stubs, gold stained, and then observed using SEM (GEMINI_SEM, Zeiss, Germany), at different magnifications, using secondary electron probes.

#### 4.4.3. Morphological Analysis—CLSM

HPLF grown on coverslips in the presence or absence of test materials for T1, T2, and T3 follow-ups were fixed with 4% paraformaldehyde in PBS for 10 min at room temperature (RT) and permeabilised with 0.1% Triton X-100 in PBS for 5 min at RT. After washing, the nonspecific binding sites were blocked with 3% BSA in PBS (blocking solution) for 10 min at RT. For double immunostaining, cells were incubated with mouse monoclonal anti-CD90 (Thy-1)/fibroblast primary antibody (Chemicon International Inc., Temecula, CA, USA), diluted 1:200 in blocking solution, O/N at 4 °C. After washings, cells were incubating with were incubated with a mixture of Alexa Fluor 488 anti-mouse IgG secondary antibody (1:2000) and Phalloidin Alexa Fluor 546 (1:300; Immunological Science, distributed by Società Italiana Chimici, Rome, Italy) in blocking solution for 30 min at RT. Internal controls of the fluorescence and functionality methodology were performed by omitting the primary antibody. Coverslips were mounted with Vectashield Mounting Medium containing DAPI (Vector Laboratories, Burlingame, CA, USA) and observed using a Leica TCS SP5 confocal microscope (Leica, Mannheim, Germany). Data for actin morphology assessment were acquired using Leica LAS AF software, and a minimum of 20 images for each determination were analysed.

## 5. Conclusions

Despite the described limitations, the obtained results indicated that all xenografts not submitted to high temperature showed important cellular stimulations, with particular and interesting interactions with the material not chemically treated. The results of this study suggest that the available alternative deproteinisation protocols are less invasive for making xenografts compatible and might improve the osteoconductive properties in regeneration interventions. Animal model experiments and randomised clinical trials are needed to confirm these results.

## Figures and Tables

**Table 1:** Dunnett’s post hoc multiple comparison results.

Dunnett’s Multiple Comparisons Test	Mean Diff.	95.00% CI of Diff.	Adjusted *p* Value
T0			
CONTROL vs. E	0.000	−0.03112 to 0.03112	>0.9999
CONTROL vs. B	0.000	−0.03112 to 0.03112	>0.9999
CONTROL vs. D	0.000	−0.03112 to 0.03112	>0.9999
CONTROL vs. G	0.000	−0.03112 to 0.03112	>0.9999
CONTROL vs. BIOS	0.000	−0.03112 to 0.03112	>0.9999
T1			
CONTROL vs. E	−0.04433	−0.08196 to −0.006706	0.0293
CONTROL vs. B	0.01500	−0.1319 to 0.1619	0.9411
CONTROL vs. D	−0.06233	−0.3709 to 0.2463	0.6323
CONTROL vs. G	−0.07700	−0.1584 to 0.004411	0.0572
CONTROL vs. BIOS	−0.06733	−0.1283 to −0.006324	0.0383
T2			
CONTROL vs. E	0.1517	−0.1327 to 0.4360	0.1765
CONTROL vs. B	0.06467	−0.01648 to 0.1458	0.0969
CONTROL vs. D	0.02300	−0.03654 to 0.08254	0.4412
CONTROL vs. G	0.07267	−0.1858 to 0.3312	0.4911
CONTROL vs. BIOS	−0.08500	−0.3450 to 0.1750	0.4022
T3			
CONTROL vs. E	0.2557	−0.1434 to 0.6547	0.1567
CONTROL vs. B	0.1223	−0.3039 to 0.5486	0.5478
CONTROL vs. D	0.009333	−0.4114 to 0.4300	0.9999
CONTROL vs. G	−0.01467	−0.4908 to 0.4614	0.9992
CONTROL vs. BIOS	0.03733	−0.3601 to 0.4348	0.9863

## Data Availability

Data can be required from the corresponding author upon reasonable request.
